# Fabrication of nanoscale T-shaped reentrant structures and its hydrophobic analysis

**DOI:** 10.1038/s41598-023-49445-y

**Published:** 2023-12-11

**Authors:** Zeping Li, Qing Wan, Jiaqi Wang, Geng Wang

**Affiliations:** 1 School of Electronic and Information Engineering, Hubei University of Science and Technology, Xianning, 437100 People’s Republic of China; 2 Key Laboratory of Photoelectric Sensing and Intelligent Control, Hubei University of Science and Technology, Xianning, 437100 Hubei People’s Republic of China; 3 School of Foreign Languages, Wuhan Institute of Technology, Wuhan, 430205 Hubei People’s Republic of China

**Keywords:** Nanoscale materials, Nanoscale biophysics

## Abstract

The present work proposes a facile method for fabricating robust hydrophobic surfaces with T-shaped reentrant nanostructures based on nano-patterning approach. The prepared surface demonstrates regularly arrangement over a large area. The hydrophobic stability of the prepared surface was analyzed theoretically using the Gibbs free energy approach, followed by being investigated experimentally. Experimental results show that the T-shaped reentrant nanostructures can significantly improve the hydrophobic stability of the surface, which is in line with the theoretical predictions. The proposed preparation method for T-shaped reentrant nanostructures provides a cost-effective and convenient way to fabricate robust hydrophobic surfaces.

## Introduction

Surface hydrophobicity, featuring high contact angles and low contact angle hysteresis, has numerous attractive practical applications such as antifouling^1–4^, self-cleaning^5^, windshield^6^, anti-fogging^7,8^, anti-icing^9^, and microfluidic control^10–13^. One method of maintaining hydrophobicity is to develop low surface tension coatings to ensure that the Cassie–Baxter (non-wetting) state remains in the minimum free energy state, but the coating is prone to surface contamination and degradation. To improve hydrophobic stability, reentrant or negative-slope structures followed by modification with fluorinated materials are developed to obtain upward net forces for lifting a droplet, even for low surface-tension liquids^14^. T-shaped reentrant structures exhibit the highest performance in liquid repellency concerning pressure balance, pinning effects, and surface curvature conditions^14^. Nanostructured reentrant surfaces represent practical advantages on the unique optical properties^15,16^, excellent pressure robustness^17,18^, modulating immune response^19^, controlling bacterial adhesion^20^, and biofilm formation^21,22^, compared with microscale structures. However, their preparation can be challenging, complicated, and costly. Despite random growth of the nanostructured surfaces is more straightforward and cost-effective, the fabricated irregular surfaces encounter less effective hydrophobicity^14^. T-shaped reentrant nanostructures have a significant influence on the wetting properties of solid surfaces.

In this report, we present a facile method for fabricating T-shaped reentrant nanostructures on mechanically stable silicon wafer based on selective etching with nano-patterning approach. The hydrophobicity robustness of the prepared T-shaped reentrant nanostructures is evaluated theoretically using the Gibbs free energy approach, followed by being investigated experimentally. Experimental results show that, the T-shaped reentrant nanostructures can significantly improve the hydrophobic stability of the surface, which is in line with our theoretical predictions.

## Results and discussion

### Surface morphology

**Figure 1:**
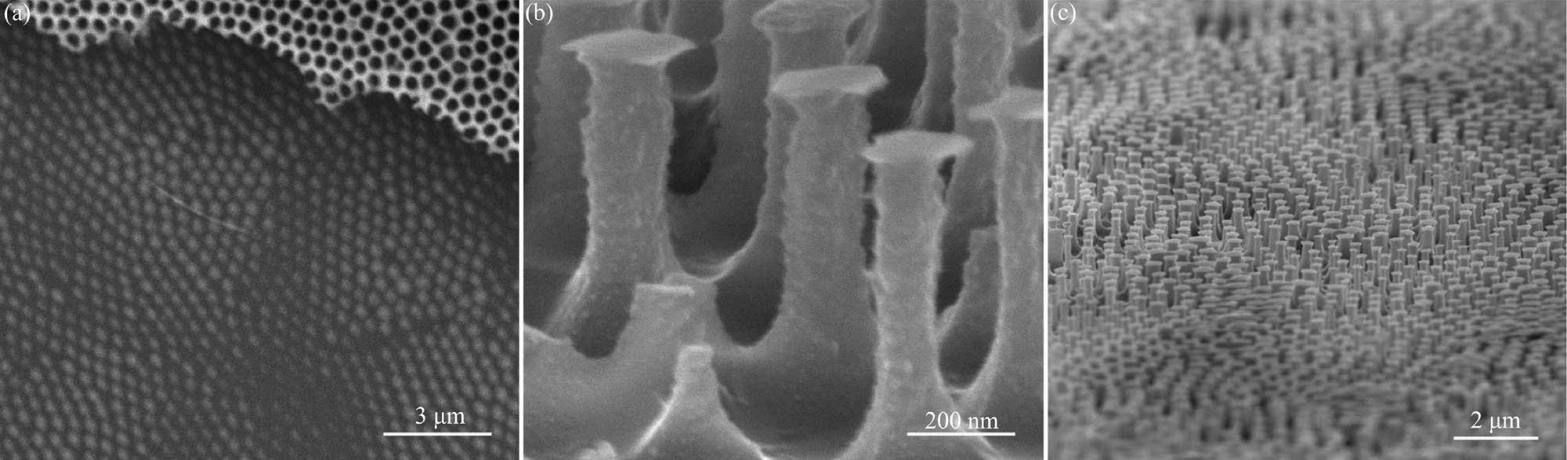
The SEM images of (**a**) Cr nanoparticles and partially unremoved AAO mask, (**b**) T-shaped reentrant nanostructures, (**c**) large-area T-shaped reentrant nanostructures.

The SEM images in Fig. 1a exhibit a highly ordered Cr nanoparticle array with a thickness of ~ 100 nm, and an intentionally retained residual AAO mask for comparison. The Cr nanoparticles were successfully and regularly deposited on the Si surface through through-hole AAO evaporation mask. Subsequently, the Si substrate was subjected to XeF_2_ isotropic etching using Cr nanoparticles as a mask to prepare T-shaped reentrant nanostructures with high aspect ratios, as shown in Fig. 1b. The SEM images in Fig. 1c display large-scale, regularly arranged T-shaped reentrant nanostructures. The geometric parameters of fabricated T-shaped reentrant nanostructures are shown in Table 1. Based on the tunable characteristics of AAO mask with tunable pore diameter and cell size and its non-lithographic properties^23^, the presented nano-patterning approach will offer attractive advantages, such as large pattern area, high throughput, low cost.

**Table 1:** The geometric parameters of fabricated T-shaped reentrant nanostructures.

D (nm)	d (nm)	H (nm)	h (nm)	P (nm)	r = r_f_ (x = H)
200	140	600	20	500	1.98

Herein r represents the roughness ratio of T-shaped reentrant nanostructures.

**Figure 2:**
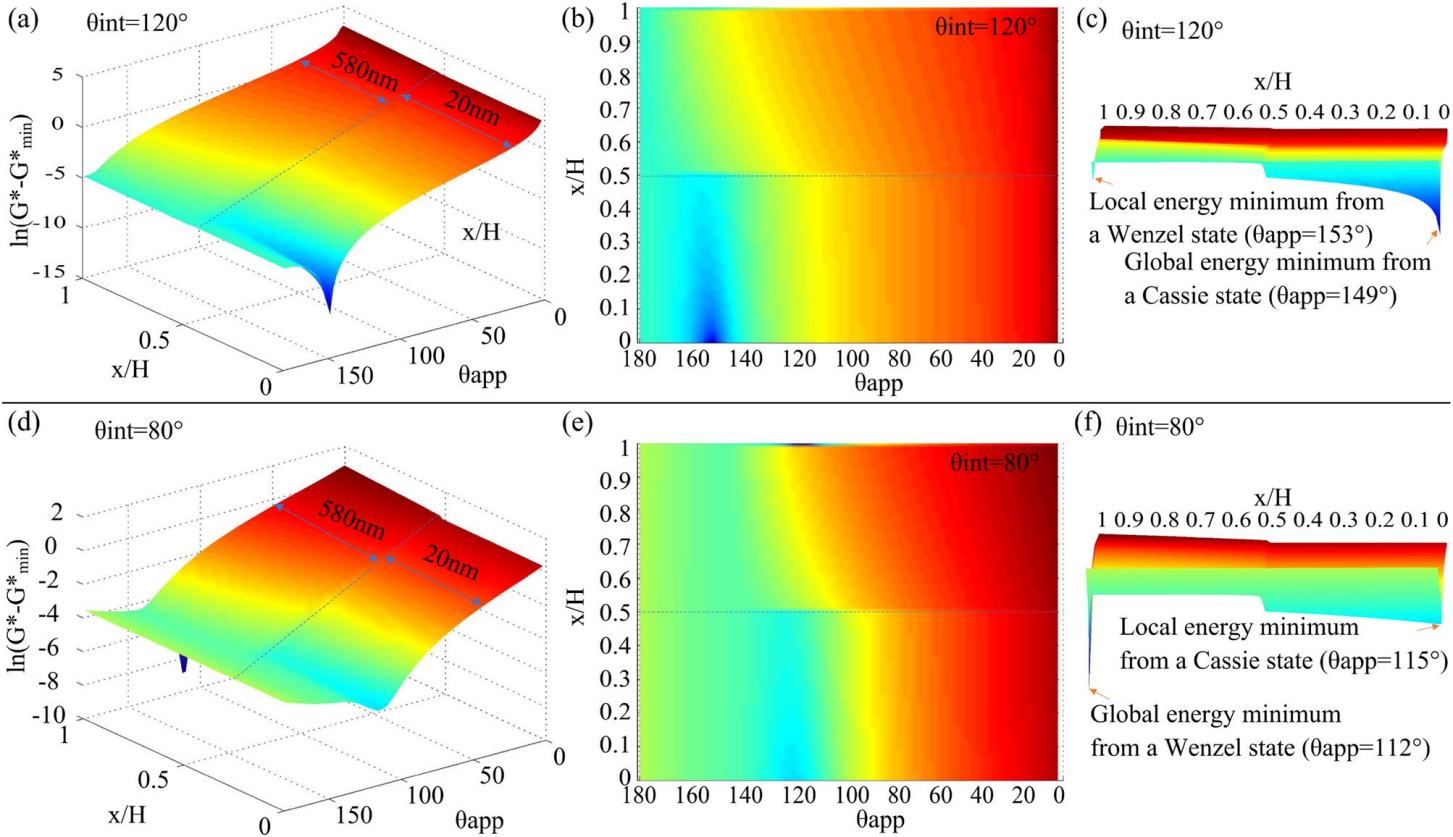
The change in the areal Gibbs free energy density. (**a**) The variation in the areal Gibbs free energy density for water propagating on a hydrophobic (θ_int_ = 120°) T-shaped reentrant nanostructured surface. (**b**) Top view of the energy diagram shown in (**a**). (**c**) Cross section view shown in (**a**). (**d**) The variation in the areal Gibbs free energy density for hexadecane propagating on an oleophilic (θ_int_ = 80°) T-shaped reentrant nanostructured surface. (**e**) Top view of the energy diagram shown in (**d**). (**f**) Cross section view shown in (**d**).

Theoretical analysis of the hydrophobic robustness. In our calculations, we assume various values for the temporary apparent contact angle (0° < θ_app_ < 180°) at a specified distance (x/H) of the liquid–air interface from the surface of T-shaped cap (normalized with respect to the height of T-shaped reentrant nanostructures H). Then the areal Gibbs free energy density (G*) of the liquid drop is calculated for each θ_app_ and x/H. In order to facilitate easier visualization of the variation in the areal Gibbs free energy density of the T-shaped reentrant nanostructured surface, an uneven scale distribution of x-axis (x/H) was adopted in Fig. 2, which is based on the height of T-shaped cap (h = 20 nm) and the height of T-shaped pillar (H–h = 580 nm).

In Fig. 2a,b, a metastable composite interface corresponding to a global minimum in free energy is observed at x ~ 0, while a fully-wetted interface corresponding to a local minimum in free energy is observed at x ~ 600 nm. The liquid droplets keep in a metastable state with relatively low energy from x ~ 0 to x ~ 20 nm. There is a potential barrier at x ~ 20 nm as shown in Fig. 2c, and the liquid droplets from x ~ 0 to x ~ 20 nm would break through the barrier after being subjected to external pressure and reach x ~ 600 nm, which is a fully-wetted interface corresponding to a local minimum in free energy.

In Fig. 2d,e, a fully-wetted interface corresponding to a global minimum in free energy is observed at x ~ 600 nm, while a metastable composite interface corresponding to a local minimum in free energy is observed at x ~ 0. The liquid droplets keep in a metastable state with relatively low energy from x ~ 0 to x ~ 20 nm. There is a potential barrier at x ~ 20 nm as shown in Fig. 2f, and the liquid droplets from x ~ 0 to x ~ 20 nm would break through the barrier after being subjected to external pressure and reach x ~ 600 nm, which is a fully-wetted interface corresponding to a global minimum in free energy.

### Surface wettability of T-shaped reentrant nanostructures

**Figure 3:**
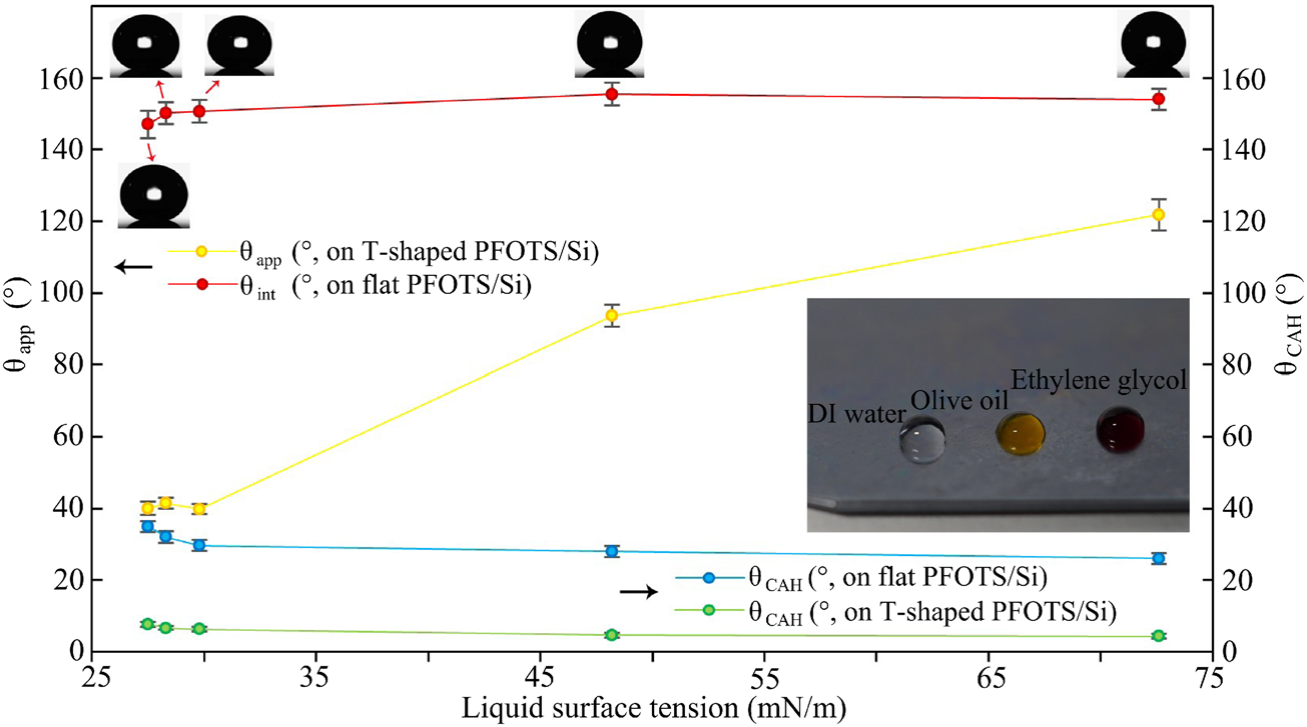
Wetting properties of the different liquids on the flat and T-shaped reentrant PFOTS/Si surface. The inset is optical image of DI water (transparent), olive oil (light yellow) and ethylene glycol (red) droplets on the T-shaped reentrant nanostructured PFOTS/Si surface.

To evaluate the wetting properties, we selected five probing liquids with different surface tensions: hexadecane, toluene, olive oil, ethylene glycol, and DI water (see Table 2). The initial examination was conducted on a flat silicon surface coated with PFOTS (as depicted in Fig. 3). The measured θ_app_ decreased with increasing liquid surface tension. Our observations showed that the apparent contact angle (θ_app_) decreased with a decrease in liquid surface tension while the transition from wetting to non-wetting (θ_app_ ≈ 90°) occurred at a critical surface tension (γ_C_) of approximately 48.2 mN/m. Furthermore, the contact angle hysteresis (θ_CAH_) remained around 20°. As expected, liquids with lower surface tensions exhibited wetting behavior on the smooth PFOTS/Si surface. In contrast, the wetting properties of the T-shaped reentrant nanostructured PFOTS/Si surface were also examined, and the results demonstrated that the surface displayed high contact angles and low contact angle hysteresis (less than 10°) for droplets of varying surface tensions. The inset shows optical image of DI water (transparent), olive oil (light yellow) and ethylene glycol (red) droplets on the T-shaped reentrant nanostructured PFOTS/Si surface. Notably, the T-shaped reentrant nanostructured surface exhibited structural colors when exposed to light.

**Table 2:** The surface tension of tested liquids measured on the flat and T-shaped reentrant PFOTS/Si surface.

Liquid	Hexadecane	Toluene	Olive oil	Ethylene glycol	DI water
γ (mN/m)	27.5	28.3	29.8	48.2	72.6

**Figure 4:**
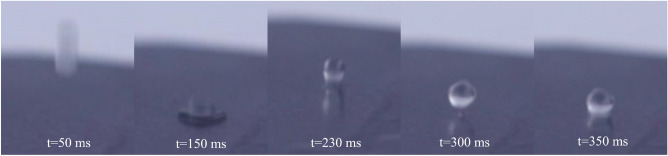
The surface of T-shaped reentrant nanostructured PFOTS/Si surface displayed the bouncing of hexadecane droplets as captured by a sequence of high-speed digital camera photograph.

Under dynamic impact conditions, the enhanced stability in withstanding the wetting transition has also been noted on the T-shaped reentrant PFOTS/Si surface. A high-speed digital camera was utilized to capture a sequence of photos, and Fig. 4 illustrates the bouncing of ethylene glycol droplets on the T-shaped reentrant PFOTS/Si surface. Following release of the ethylene glycol droplets, they immediately contacted with PFOTS/Si’s surface and spread out there. The droplet then contracted, fully rebounded, and detached from the PFOTS/Si surface before stabilizing there (Fig. 4, Supplemental Material, Movie 1).

## Conclusions

In summary, we proposed a facile method to fabricate robust hydrophobic surfaces with T-shaped reentrant nanostructures. The prepared T-shaped reentrant nanostructures with regularly arrangement in a large area were demonstrated and the hydrophobic stability of the prepared surface was analyzed theoretically using the Gibbs free energy approach and experimentally. Experimental results show that the T-shaped reentrant nanostructures can significantly improve the hydrophobic stability of the surface, which is in line with the theoretical predictions. The proposed preparation method for T-shaped reentrant nanostructures provides a cost-effective and convenient way to fabricate robust hydrophobic surfaces.

## Methods

### Fabrication of T-shaped reentrant nanostructures

**Figure 5:**
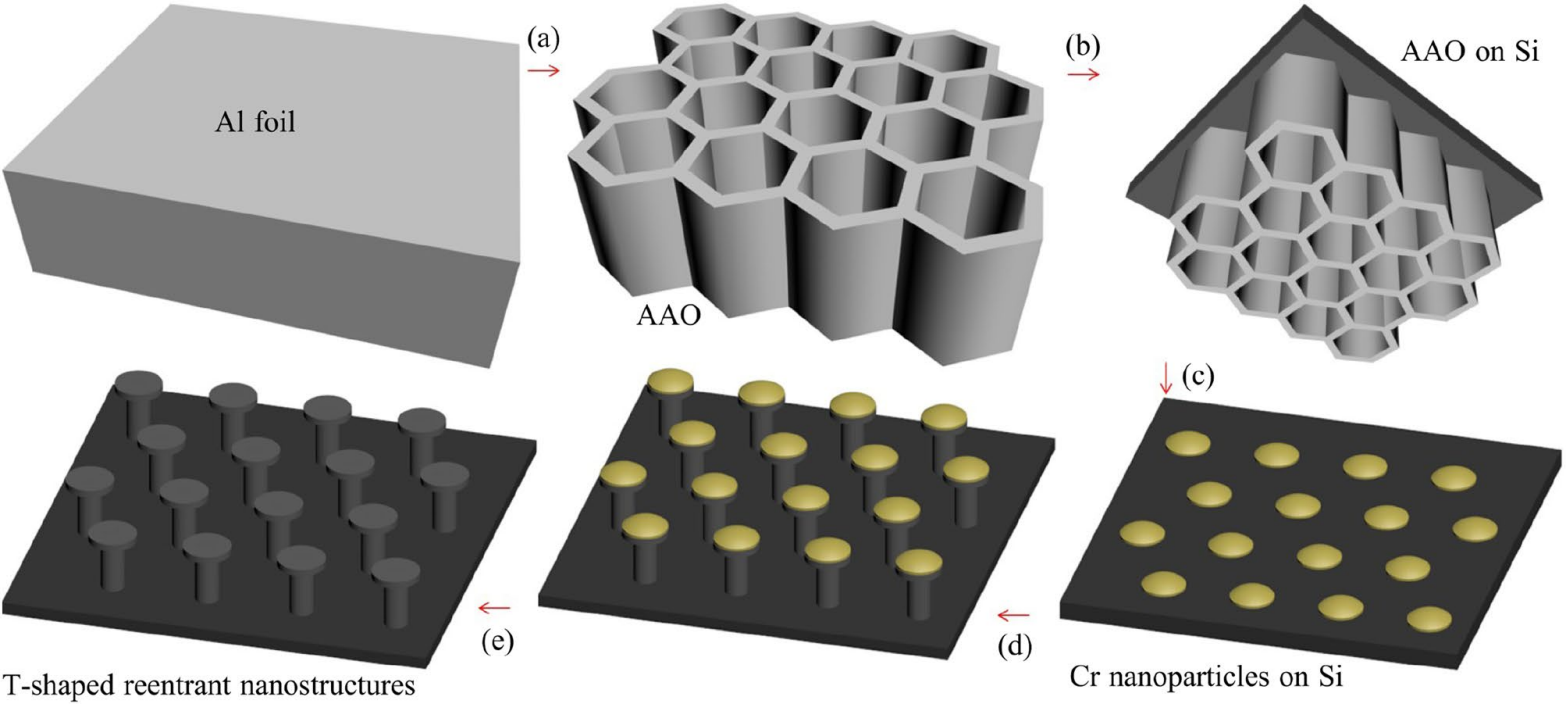
Schematic diagram of the fabrication process for T-shaped reentrant nanostructures. (**a**) Two-step anodization, (**b**) through-hole AAO membrane fixed on a Si substrate as an evaporation mask, (**c**) depositing of the Cr nanoparticle array on the Si substrate, (**d**) selective XeF_2_ isotropic etching of Si substrate using Cr nanoparticles as a mask, (**e**) removing Cr nanoparticles.

The through-hole anodic aluminium oxide (AAO) membranes were prepared by the well-known two-step anodization process (Fig. 5a) as our previous work^24^. The first anodization was carried out in 1% phosphoric acid and 0.01 M aluminum oxalate hydrate for 4 h at the temperature of 1 °C and voltage of 195 V. Perform a second anodization for 2.5 h under the same conditions. Subsequently, the pore-widening and pore-opening process was finished in 5% phosphoric acid solution for 50 min at 50 °C. The prepared AAO membranes were transferred onto the Si substrates in De-ionized (DI) water for keeping the membranes from twisting, folding and cracking (Fig. 5b). To achieve good mechanical robustness, a silicon wafer with the high Young’s modulus of Si (up to 66 GPa) is employed to obtain T-shaped reentrant nanostructure. Subsequently, an array of Cr nanoparticles with a thickness of approximately 50 nm was deposited onto the Si surface via AAO pores using electron beam evaporation (Fig. 5c). Then, the AAO membranes were removed with a commercial tape. The resulting Cr nanoparticle array on the Si substrates would serve as the subsequent etching template. The selective XeF_2_ isotropic etching was employed to obtain T-shaped reentrant nanostructure on the Si substrate (Fig. 5d). Finally, a T-shaped reentrant nanostructure was formed after removing Cr nanoparticles from the Si surface (Fig. 5e). Despite its reentrant structure, the surface must still undergo chemical modification for increased resistance to wetting, then samples were treated with 1H, 1H, 2H, 2H-perfluoro-octyltrichlorosilane (PFOTS, Sigma–Aldrich) in the vapor phase at 140 °C for 5 min^25^.

### Theoretical modeling and simulation

**Figure 6:**
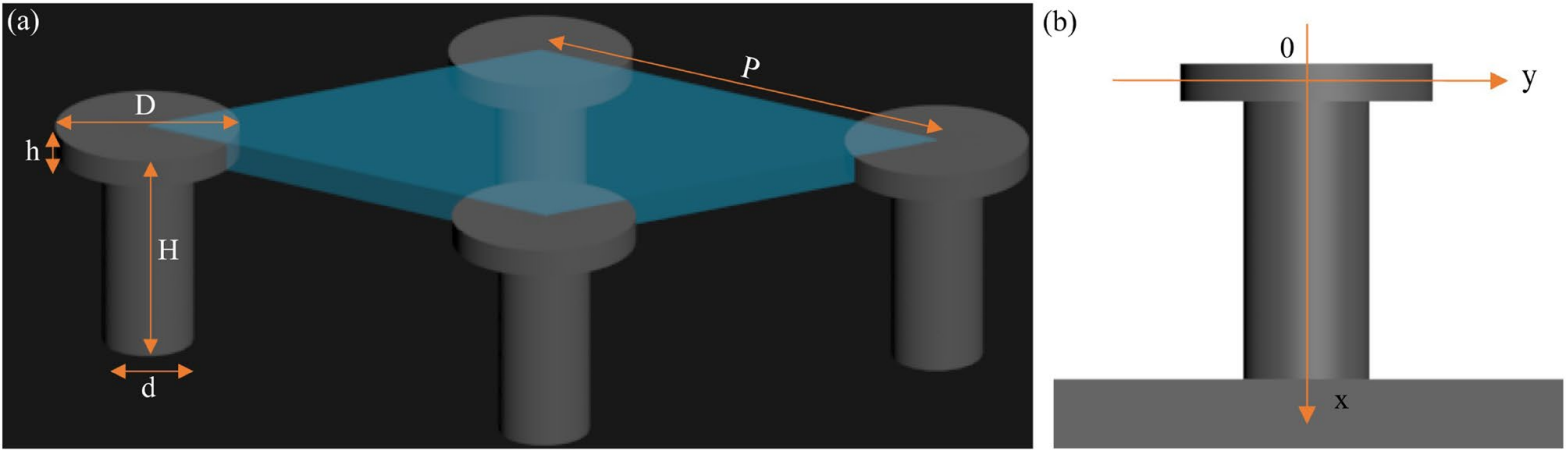
A schematic illustration of the topological profile in the calculation of the change in the Gibbs free energy density on the propagation of the liquid–air interface. (**a**) Geometric parameters in a discrete unit (blue area) with hexagonal arrangement, (**b**) the coordinate system used in the calculation. Herein D represents the diameter of T-shaped cap, d represents the diameter of T-shaped pillar, H represents the height of T-shaped reentrant nanostructures, h represents the height of T-shaped cap, P represents the pattern pitch of T-shaped reentrant nanostructures.

A theoretical modeling of the wetting process, based on Marmur’s^26^ and Tuteja’s works^25^, was used to calculate the change in the Gibbs free energy density with the evolution of the solid–liquid interface for evaluating the stability of a composite interface on the T-shaped reentrant surface. Based on geometric parameters in Fig. 6a and the coordinate system in Fig. 6b, the formulations of geometric parameters are built as shown in Table 3.

**Table 3:** Formulations of geometric parameters for the hexagonal arrangement.

Parameters	0 ≤ x ≤ h	h < x < H	x = H
r_f_	1 + 4x/D	2D^2^/d^2^ + 4Dh/d^2^ + 4x/d1	[$\sqrt {3}$/2(Pd)^2^ + πD^2^/2πd^2^/4 + πDh + πd(H–h)]/($\sqrt {3}$/2P^2^)
f_s_	πD^2^/(2$\sqrt {3}$P^2^)	πd^2^/(2$\sqrt {3}$P^2^)	1

Herein r_f_ is the roughness of wetted solid, defined as the actual wetted surface area divided by its projected surface area, f_s_ is the solid fraction, defined as the projected wetted surface area divided by the nominal surface area, θ_int_ is the intrinsic contact angle on the smooth and flat surface, θ_app_ is the apparent contact angle, G* is the areal Gibbs free energy density as a function of θ_app_ and x/H.

Based on the theoretical modeling, a Matlab® (Mathworks Inc.) code was developed, and the areal Gibbs free energy density (G*) variation of the liquid drop was computed for water (γ_lv_ = 72.6 mN/m, θ = 120°) and hexadecane (γ_lv_ = 27.5 mN/m, θ = 80°). Herein γ is the surface tension, and subscripts s, v, and l represent solid, vapor and liquid, respectively.

From the thermodynamic perspective, the areal Gibbs free energy density of a given volume of liquid droplet at equilibrium on a substrate is given by the following equation

1 $$G^{*} = (2 - 3\cos \theta_{app} + \cos^{3} \theta {}_{app})^{ - 2/3} \left[ {{2} - {2}\cos \theta_{app} - \sin^{2} \theta_{app} (r_{f} f_{s} \cos \theta_{{\text{int}}} + f_{s} - 1)} \right].$$

### Sample characterization

The fabricated Cr nanoparticles, unremoved AAO mask and T-shaped reentrant nanostructures were observed and their images were taken using field emission scanning electron microscopy (FESEM, FEI Nova NanoSEM 450). The liquid-repellent properties of liquid droplets on the sample surfaces were characterized using an optical contact-angle system (OCA20, Dataphysics, Germany). DI water of ∼ 5 μL and hexadecane (Sigma-Aldrich) drops of ∼ 2 μL were used as CA test solvents. To accurately express the wettability of the surface, the contact angle with 3–5 different positions on the surface was measured.

### Supplementary Information

Supplementary Movie 1.

## Data Availability

The data used to support the findings of this study are available from the corresponding author upon request.
